# Non-persistence with multiple secondary prevention medications for peripheral arterial disease among older hypertensive patients

**DOI:** 10.3389/fphar.2024.1464689

**Published:** 2024-12-18

**Authors:** Martin Wawruch, Miriam Petrova, Denisa Celovska, Sofa D. Alfian, Tomas Tesar, Jan Murin, Michal Trnka, Tomas Paduch, Emma Aarnio

**Affiliations:** ^1^ Institute of Pharmacology and Clinical Pharmacology, Faculty of Medicine, Comenius University, Bratislava, Slovakia; ^2^ Department of Internal Medicine, Faculty of Medicine, Comenius University, Bratislava, Slovakia; ^3^ Department of Pharmacology and Clinical Pharmacy, Faculty of Pharmacy, Universitas Padjadjaran, Bandung, Indonesia; ^4^ Center of Excellence in Higher Education for Pharmaceutical Care Innovation, Universitas Padjadjaran, Bandung, Indonesia; ^5^ Department of Organisation and Management of Pharmacy, Faculty of Pharmacy, Comenius University, Bratislava, Slovakia; ^6^ Institute of Medical Physics and Biophysics, Faculty of Medicine, Comenius University, Bratislava, Slovakia; ^7^ 2nd Department of Surgery, Center for Vascular Disease, St. Anne’s University Hospital and Faculty of Medicine, Masaryk University, Brno, Czechia; ^8^ School of Pharmacy, University of Eastern Finland, Kuopio, Finland

**Keywords:** peripheral arterial disease, non-adherence, non-persistence, new user, general practitioner, statins, antiplatelet agents, angiotensin-converting enzyme inhibitors

## Abstract

**Introduction:**

The benefit of secondary prevention in hypertensive patients with peripheral arterial disease (PAD) is based on continual simultaneous taking of statins, antiplatelet agents and antihypertensive agents, preferably angiotensin-converting enzyme inhibitors (ACEIs) or angiotensin receptor blockers (ARBs). Our study was aimed at a) the analysis of the extent of non-persistence with multiple medication classes, and b) identifying factors associated with the likelihood of non-persistence.

**Methods:**

In our cohort study, 3,401 hypertensive patients (1,853 females and 1,548 males) aged ≥65 years treated simultaneously with statins, antiplatelet agents and ACEIs/ARBs and in whom PAD was newly diagnosed during 2012 were analysed. A patient was classified as non-persistent when he/she was non-persistent with at least one of the three analysed medication classes. The most important characteristics associated with the probability of non-persistence were identified using the Cox regression.

**Results:**

At the end of the follow-up period (mean length 1.8 years), 1,869 (55.0%) patients (including 1,090 females and 779 males) were classified as non-persistent. In the whole study cohort, factors associated with non-persistence were female sex, atrial fibrillation, and being a new user of at least one of the analysed medication classes; in males, they were university education, atrial fibrillation, and epilepsy, and, in females, being a new user.

**Conclusion:**

Identification of sex differences in factors associated with non-persistence makes it possible to determine the groups of patients in whom special attention should be paid to improving their persistence with a combination of medicines in order to ensure successful secondary prevention of PAD.

## 1 Introduction

Peripheral arterial disease (PAD) is caused by narrowing of arteries of lower limbs which is based mostly on accumulation of atherosclerotic plaques in arteries. Lower extremity PAD may lead to limb-related complications like intermittent claudication, ischemic rest pain, ischemic ulcer and gangrene requiring amputation. More than 50% of PAD patients are asymptomatic (Bevan and White Solaru, 2020; Shamaki et al., 2022). According to the Global Burden of Diseases of 2017, the worldwide number of prevalent cases of PAD was 118.1 million and the number of incident cases was 10.8 million (GBD, 2017 disease and injury incidence and prevalence collaborators, 2017; Guidelines for ATC Classification and DDD Assignment, 2018, 2018). PAD is associated with an increased risk of developing coronary artery disease and cerebrovascular disease and of cardiovascular (CV) death (Shamaki et al., 2022). The prevalence of PAD is increasing with an advancing age. According to the study by Kalbaugh et al. (2017), the annual prevalence of PAD ranged from 12.5% in 2003 to 18.5% in 2012 among patients aged ≥75 years, while in patients aged 65–74 years it ranged from 8.0% to 9.0% over the same time frame.

Risk factors of PAD include smoking, diabetes mellitus, hyperlipidaemia, arterial hypertension, chronic kidney disease and a family history of vascular disease. Conservative treatment of PAD includes treatment of modifiable risk factors of PAD (e.g., smoking cessation, antihypertensive and antidiabetic treatment) and administration of secondary preventive medications, namely, statins and antiplatelet agents (Aboyans et al., 2018; Bevan and White Solaru, 2020; Firnhaber and Powell, 2019; Gornik et al., 2024).

Arterial hypertension is one of the most important modifiable risk factors of PAD. In 35%–55% of patients with PAD, hypertension can be found when PAD is diagnosed (Clement et al., 2004). Two randomised clinical trials provided direct evidence to support administration of angiotensin-converting enzyme inhibitors (ACEIs)/angiotensin receptor blockers (ARBs) for blood pressure control among PAD patients (Sleight, 2000; Yusuf et al., 2008). In PAD patients with arterial hypertension, administration of ACEIs/ARBs should be considered as the first line treatment of arterial hypertension (Aboyans et al., 2018). For these patients, administration of ACEIs/ARBs is recommended in order to reduce the risk of major adverse CV events (MACE) including myocardial infarction (MI), stroke, heart failure and CV death (Armstrong et al., 2015; Gornik et al., 2024; Piller et al., 2014; Sleight, 2000; Yusuf et al., 2008).

Adherence to medication is a necessary precondition of successful secondary prevention in PAD patients. Adherence to medications characterises the process by which patients take their medications as prescribed. Adherence includes three interrelated phases: initiation, implementation and persistence (Vrijens et al., 2012). Initiation represents the taking of the first dose of medication. Implementation reflects the extent to which a patient´s dosing corresponds to the prescribed dosing regimen, from initiation until the last dose is taken. Persistence characterises the length of time between initiation and the last dose before discontinuation. Medication non-adherence may occur in any of these phases, e.g., as non-initiation, suboptimal implementation of the dosing regimen (late, skipped, reduced or extra doses, drug holidays) or early discontinuation (non-persistence) (De Geest et al., 2018).

Older age has been reported as a factor associated with non-persistence in several studies focused on CV medications (Alfian et al., 2019a; Chen et al., 2019; Kim et al., 2021). For this reason, our study analysed non-persistence in the subgroup of older patients. Medication-related factors (e.g., increase in the number of medications, increase in the number and severity of adverse effects, cost) and patient-related factors (e.g., vision loss, low cognition skills, forgetfulness, depression, belief that the medicine is not needed) represent reasons for non-adherence in older patients (Doggrell, 2010). Our study was aimed at a) the analysis of the extent of non-persistence with multiple medications (statins, antiplatelet agents and ACEIs/ARBs) among older hypertensive PAD patients, and b) identifying factors associated with the likelihood of non-persistence in the whole study cohort and separately among males and females. Our study is particularly important because proper and regular administration of secondary preventive medications is necessary for achieving the treatment effect consisting in reducing the development of limb-related complications and MACE in PAD patients. Since the treatment of PAD requires a combination of medications, analysing persistence simultaneously with all three medication classes gives a better picture of true adherence than analysing persistence with each medication class separately.

## 2 Materials and methods

### 2.1 Database and study population

**FIGURE 1:**
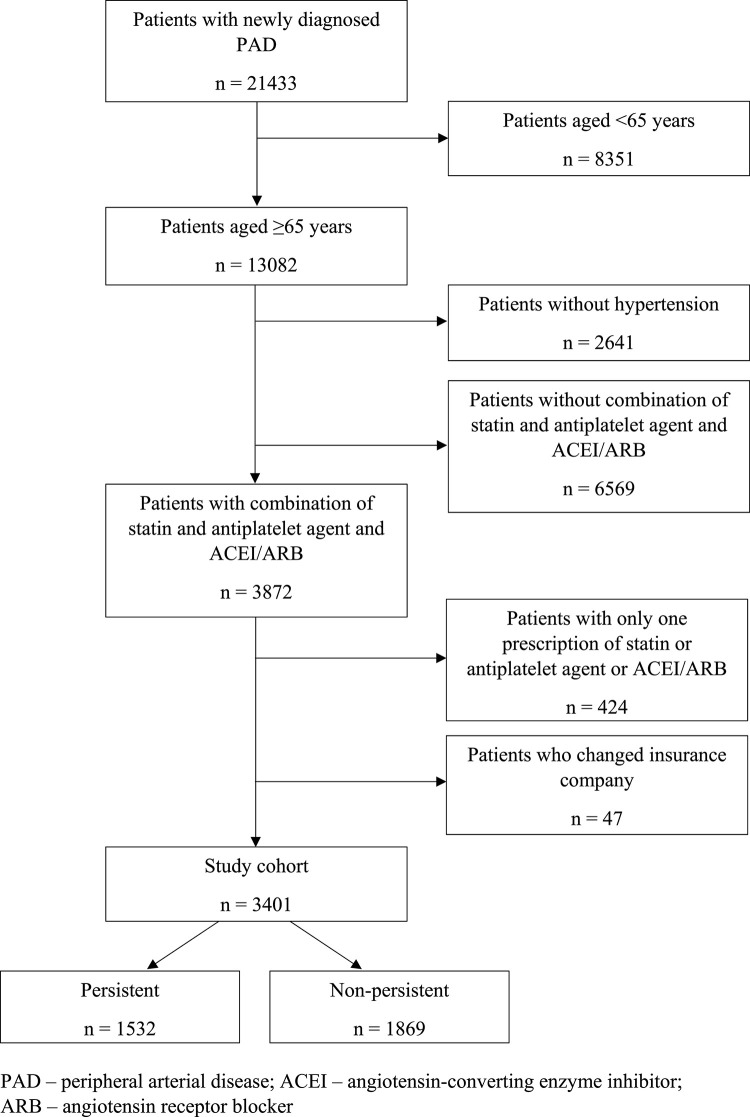
Flow chart showing derivation of the study cohort (n = 3,401).

Our study cohort (n = 3,401) was derived from a group of 21,433 patients in whom PAD was newly diagnosed between 1 January and 31 December 2012. Of this group, 13,082 patients aged ≥65 years were selected. After exclusion of 2,641 patients without arterial hypertension and those without a combination of statin, antiplatelet agent and ACEI/ARB (n = 6,569), there remained a sample of 3,872 older patients with a combination of statin, antiplatelet agent and ACEI/ARB. After exclusion of 424 patients with only a single prescription for statin, antiplatelet agent or ACEI/ARB, and 47 patients who changed their health insurance company during the follow-up period, there remained a sample of 3,401 patients which represented the study cohort for our analyses (Figure 1). Patients with only one prescription for statin, antiplatelet agent or ACEI/ARB were excluded because a single prescription of these medications may be a consequence of adverse effects or intolerance rather than non-persistence. Our study cohort was selected from the database of the General Health Insurance Company which is the largest health insurance provider in the Slovak Republic covering approximately 63% of the population. Our study population may be considered as representative since older and sicker patients, in whom the prevalence of PAD is relatively higher compared to younger population, are insured mostly through the General Health Insurance Company.

### 2.2 Analysis of non-persistence

There are several methods to evaluate persistence with multiple medications (Alfian et al., 2019b). First, measuring persistence with “all medications” in which patients are defined as non-persistent when at least one medication has a treatment gap. Second, measuring persistence with “both medications” in which patients are defined as non-persistent when there is a treatment gap in days with both medication A and medication B. Third, measuring persistence with any medication in which patients are defined as non-persistent when there is a treatment gap for all medications, i.e., no medication in the treatment gap period. Since, in order to benefit from the treatment, the patient is to take all recommended medications, represented in our study by a combination of statins, antiplatelet medications, and ACEIs/ARBs, we decided to use the first above-mentioned method.

In our retrospective cohort study, a patient was classified as non-persistent when he/she was non-persistent with at least one of the three analysed medication groups (statins, antiplatelet agents, ACEIs/ARBs). Non-persistence with medication groups mentioned above was identified similarly as in previous studies that focused on separate evaluation of non-persistence with each individual medication class (Wawruch et al., 2019; Wawruch et al., 2021; Wawruch et al., 2022). In these separate analyses, a patient was identified as non-persistent if there was an at least 6-month treatment gap without a prescription for the particular medication. Treatment gaps were observed after the estimated last day covered by the last package of prescribed medication. In the study presented in this manuscript, the length of treatment before treatment gap was identified according to the first medication in which non-persistence occurred. The dosage of medications was not taken into consideration.

The index date was the date of the first prescription of analysed medication classes (statin, antiplatelet agent, ACEI/ARB) after PAD was diagnosed. Patients were followed from the index date until the end of the five-year follow-up period, until non-persistence with at least one of the analysed medication classes, or until death, which ever occurred first. Censoring was applied in patients who died to prevent their being incorrectly classified as non-persistent.

### 2.3 Factors associated with non-persistence

Following factors were analysed as characteristics potentially associated with non-persistence:

a) Socio-demographic characteristics: age, sex, university education, and employment;
b) History of CV events: stroke, transient ischemic attack and MI;
c) Number of comorbid conditions and particular comorbidities identified according to the 10th revision of the International Classification of Diseases (ICD, 1992);
d) Medication-related characteristics:
- whether a patient was a new user of any of the analysed medication classes (statins, antiplatelet agents, ACEIs/ARBs), meaning that treatment with a particular medication class was initiated in association with the diagnosis of PAD;
- patient’s co-payment calculated as a sum of co-payments for the particular analysed medications (statins, antiplatelet agents, ACEIs/ARBs);
- whether the initial prescriber of any of the analysed medication classes was a general practitioner;
e) The overall number of medications and the number of CV medications recorded for a patient, and particular classes of CV medications identified according to the ATC classification (Guidelines for ATC Classification and DDD Assignment, 2018, 2018).

### 2.4 Statistical analyses

Continuous variables were expressed as means ± standard deviations and categorical variables as frequencies and percentages.

Categorical variables were compared between the two groups using the χ^2^-test. The comparison of continuous variables between the two groups was performed using the Mann-Whitney U test. This non-parametric test was applied because of non-Gaussian distribution of evaluated variables. The normality of distribution of continuous variables was assessed by the Kolmogorov-Smirnov test.

The Kaplan-Meier model was applied to compare persistence during the 5-year follow-up period between males and females. The log-rank test was used to identify statistically significant difference in the development of persistence between sexes. The Cox proportional hazards model was applied to identify the most important characteristics associated with the probability of non-persistence. For each characteristics the hazard ratio and corresponding 95% confidence interval were calculated. In the Cox model, the time under treatment was considered to be the time under treatment of the medication belonging to one of the three analysed medication classes in which the earliest discontinuation occurred. Because of a significant difference in the development of persistence between sexes in the Kaplan-Meier model we performed Cox regression analyses in the whole study cohort and separately in males and females (Newman, 2001). To evaluate the explanatory power of particular Cox models, the Harrell´s C-index was calculated. The closer the value of this index is to 1, the better explanatory power the model is deemed to have (Harrell et al., 1996; Uno et al., 2011).

All statistical tests were carried out at the level of significance of α = 0.05. The statistical software IBM SPSS for Windows, version 29, was used (IBM SPSS Inc., Armonk, NY, United States).

## 3 Results

Our study cohort consisted of 3,401 PAD patients aged ≥65 years (1,853 females and 1,548 males) in whom simultaneous administration of statins, antiplatelet agents and ACEIs/ARBs was recorded. Table 1 shows the baseline characteristics of the study cohort.

**TABLE 1:** Baseline characteristics of the study cohort.

Factor	The whole study cohort				Males				Females			
	All (n = 3,401)	Persistent (n = 1,532)	Non-persistent (n = 1,869)	p	All (n = 1,548)	Persistent (n = 769)	Non-persistent (n = 779)	p	All (n = 1,853)	Persistent (n = 763)	Non-persistent (n = 1,090)	p
Socio-demographic characteristics												
Age	74.3 ± 6.3	75.2 ± 6.7	73.5 ± 5.8	<0.001*	73.2 ± 6.0	73.7 ± 6.3	72.7 ± 5.7	0.007*	75.1 ± 6.3	76.7 ± 6.7	74.0 ± 5.8	<0.001*
Female sex	1853 (54.5)	763 (49.8)	1,090 (58.3)	**<0.001**								
University education	260 (7.6)	101 (6.6)	159 (8.5)	**0.037**	200 (12.9)	79 (10.3)	121 (15.5)	**0.002**	60 (3.2)	22 (2.9)	38 (3.5)	0.471
Employed patients	172 (5.1)	71 (4.6)	101 (5.4)	0.308	122 (7.9)	53 (6.9)	69 (8.9)	0.151	50 (2.7)	18 (2.4)	32 (2.9)	0.451
History of CV events^a^												
History of ischemic stroke	683 (20.1)	352 (23.0)	331 (17.7)	**<0.001**	325 (21.0)	193 (25.1)	132 (16.9)	**<0.001**	358 (19.3)	159 (20.8)	199 (18.3)	0.166
History of TIA	261 (7.7)	116 (7.6)	145 (7.8)	0.839	94 (6.1)	46 (6.0)	48 (6.2)	0.882	167 (9.0)	70 (9.2)	97 (8.9)	0.839
History of MI	287 (8.4)	171 (11.2)	116 (6.2)	**<0.001**	143 (9.2)	92 (12.0)	51 (6.5)	**<0.001**	144 (7.8)	79 (10.4)	65 (6.0)	**<0.001**
Comorbid conditions												
Number of comorbid conditions	2.9 ± 1.6	3.0 ± 1.6	2.8 ± 1.5	<0.001*	2.6 ± 1.5	2.9 ± 1.5	2.4 ± 1.5	<0.001*	3.1 ± 1.6	3.2 ± 1.6	3.0 ± 1.5	0.088*
Chronic heart failure	277 (8.1)	157 (10.2)	120 (6.4)	**<0.001**	115 (7.4)	73 (9.5)	42 (5.4)	**0.002**	162 (8.7)	84 (11.0)	78 (7.2)	**0.004**
Atrial fibrillation	406 (11.9)	189 (12.3)	217 (11.6)	0.516	181 (11.7)	90 (11.7)	91 (11.7)	0.989	225 (12.1)	99 (13.0)	126 (11.6)	0.359
Diabetes mellitus	1,573 (46.3)	773 (50.5)	800 (42.8)	**<0.001**	693 (44.8)	373 (48.5)	320 (41.1)	**0.003**	880 (47.5)	400 (52.4)	480 (44.0)	**<0.001**
Hypercholesterolemia	1,577 (46.4)	712 (46.5)	865 (46.3)	0.910	664 (42.9)	360 (46.8)	304 (39.0)	**0.002**	913 (49.3)	352 (46.1)	561 (51.5)	**0.024**
Dementia	255 (7.5)	144 (9.4)	111 (5.9)	**<0.001**	98 (6.3)	61 (7.9)	37 (4.7)	**0.010**	157 (8.5)	83 (10.9)	74 (6.8)	**0.002**
Depression	398 (11.7)	186 (12.1)	212 (11.3)	0.471	102 (6.6)	62 (8.1)	40 (5.1)	**0.020**	296 (16.0)	124 (16.3)	172 (15.8)	0.785
Anxiety disorders	1,029 (30.3)	452 (29.5)	577 (30.9)	0.387	302 (19.5)	162 (21.1)	140 (18.0)	0.124	727 (39.2)	290 (38.0)	437 (40.1)	0.366
Parkinson’s disease	147 (4.3)	83 (5.4)	64 (3.4)	**0.004**	55 (3.6)	35 (4.6)	20 (2.6)	**0.035**	92 (5.0)	48 (6.3)	44 (4.0)	**0.028**
Epilepsy	109 (3.2)	52 (3.4)	57 (3.0)	0.570	54 (3.5)	24 (3.1)	30 (3.9)	0.434	55 (3.0)	28 (3.7)	27 (2.5)	0.137
Bronchial asthma/COPD	672 (19.8)	309 (20.2)	363 (19.4)	0.586	314 (20.3)	172 (22.4)	142 (18.2)	**0.043**	358 (19.3)	137 (18.0)	221 (20.3)	0.213
CV co-medication												
Number of medications	8.5 ± 2.3	8.7 ± 2.2	8.4 ± 2.5	<0.001*	8.2 ± 2.6	8.6 ± 2.3	7.8 ± 2.8	<0.001*	8.8 ± 2.1	8.9 ± 2.1	8.7 ± 2.2	0.149*
Number of CV medications	5.4 ± 2.1	5.6 ± 2.1	5.2 ± 2.0	<0.001*	5.1 ± 2.0	5.4 ± 2.1	4.8 ± 1.9	<0.001*	5.6 ± 2.1	5.8 ± 2.2	5.5 ± 2.0	0.008*
Cardiac glycosides	211 (6.2)	125 (8.2)	86 (4.6)	**<0.001**	75 (4.8)	52 (6.8)	23 (3.0)	**<0.001**	136 (7.3)	73 (9.6)	63 (5.8)	**0.002**
Antiarrhythmic agents	240 (7.1)	119 (7.8)	121 (6.5)	0.143	113 (7.3)	61 (7.9)	52 (6.7)	0.342	127 (6.9)	58 (7.6)	69 (6.3)	0.286
Beta-blockers	711 (20.9)	355 (23.2)	356 (19.0)	**0.003**	295 (19.1)	173 (22.5)	122 (15.7)	**<0.001**	416 (22.5)	182 (23.9)	234 (21.5)	0.226
Loop diuretics	761 (22.4)	409 (26.7)	352 (18.8)	**<0.001**	306 (19.8)	190 (24.7)	116 (14.9)	**<0.001**	455 (24.6)	219 (28.7)	236 (21.7)	**<0.001**
Thiazide diuretics	814 (23.9)	373 (24.3)	441 (23.6)	0.609	302 (19.5)	162 (21.1)	140 (18.0)	0.124	512 (27.6)	211 (27.7)	301 (27.6)	0.985
Mineralocorticoid receptor antagonists	259 (7.6)	166 (10.8)	93 (5.0)	**<0.001**	122 (7.9)	83 (10.8)	39 (5.0)	**<0.001**	137 (7.4)	83 (10.9)	54 (5.0)	**<0.001**
Calcium channel blockers	1,058 (31.1)	496 (32.4)	562 (30.1)	0.148	416 (26.9)	216 (28.1)	200 (25.7)	0.284	642 (34.6)	280 (36.7)	362 (33.2)	0.121
Anticoagulants	694 (20.4)	343 (22.4)	351 (18.8)	**0.009**	303 (19.6)	167 (21.7)	136 (17.5)	**0.035**	391 (21.1)	176 (23.1)	215 (19.7)	0.083
Lipid-lowering agents other than statins^b^	330 (9.7)	142 (9.3)	188 (10.1)	0.439	143 (9.2)	76 (9.9)	67 (8.6)	0.384	187 (10.1)	66 (8.7)	121 (11.1)	0.085
Study medication-related characteristics												
Patient´s co-payment (EUR)^c^	5.7 ± 4.1	5.6 ± 4.1	5.7 ± 4.1	0.325*	5.7 ± 4.1	5.7 ± 4.0	5.8 ± 4.1	0.646*	5.6 ± 4.1	5.5 ± 4.1	5.7 ± 4.1	0.303*
New user^d^	745 (21.9)	309 (20.2)	436 (23.3)	**0.027**	359 (23.2)	160 (20.8)	199 (25.5)	**0.027**	386 (20.8)	149 (19.5)	237 (21.7)	0.248
General practitioner as index prescriber^e^	3,134 (92.1)	1,423 (92.9)	1,711 (91.5)	0.149	1,428 (92.2)	725 (94.3)	703 (90.2)	**0.003**	1,706 (92.1)	698 (91.5)	1,008 (92.5)	0.435

In the case of categorical variables, values represent the frequency and the percentages are provided in parentheses (% of n). In the case of continuous variables, means ± standard deviations are provided. CV, cardiovascular; TIA, transient ischemic attack; MI, myocardial infarction; COPD, chronic obstructive pulmonary disease; p–statistical significance between persistent and non-persistent patients according to the χ^2^-test; * statistical significance according to the Mann-Whitney U test; in the case of statistical significance (p< 0.05), the values are expressed in bold.

^a^ The time period covered by “history” – 5 years before the index date of this study.

^b^ Lipid lowering agents other than statins–ezetimibe and fibrates.

^c^ Co-payment–calculated as a sum of co-payments of particular analysed medications (statins, antiplatelet agents, ACEIs/ARBs) paid by the patient per month.

^d^ New user–patient in whom at least one of the analysed medications (statins, antiplatelet agents, ACEIs/ARBs) was initiated in association with the diagnosis of peripheral arterial disease.

^e^ General practitioner as index prescriber–whether an initial prescriber of any of the analysed medication classes (statins, antiplatelet agents, ACEIs/ARBs) was a general practitioner.

**FIGURE 2:**
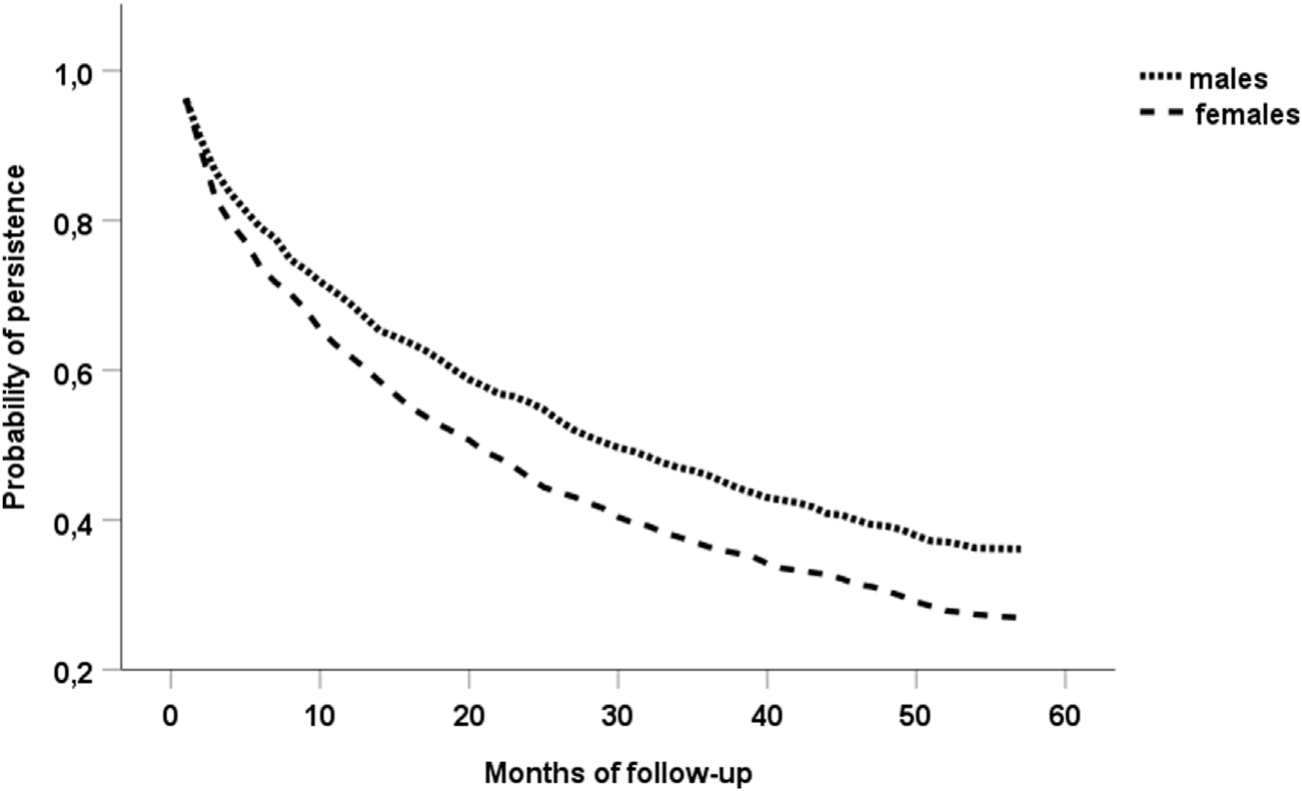
Persistence among the groups of males and females.

In the whole study cohort, non-persistence with at least one of the analysed medication classes (statins, antiplatelet agents, ACEIs/ARBs) was found during the 1st, 2nd, 3rd, 4th and 5th year of the follow-up period in 1,054 (31.0%), 391 (11.5%), 222 (6.5%), 132 (3.9%), and 70 (2.1%) patients, respectively. During those years of the follow-up period, 423 (27.3%), 162 (10.5%), 100 (6.5%), 66 (4.2%), and 28 (1.8%) males, respectively, became non-persistent. Similarly, 631 (34.0%), 229 (12.3%), 122 (6.6%), 66 (3.6%), and 42 (2.3%) females, respectively, became non-persistent during individual years of the follow-up period. At the end of the follow-up period (mean length 1.8 years), 1,869 (55.0%) patients were non-persistent with at least one of the analysed medication classes. A significantly higher proportion of females was found to be non-persistent with at least one of the three medication classes at the end of the follow-up (1,090 (58.8% of 1,853 females) vs. 779 (50.3% of 1,548 males), p< 0.001 according to the χ^2^-test). The sharper decline of the survival curve of females in the Kaplan-Meier model also indicated a significantly higher proportion of females becoming non-persistent during the follow-up in comparison with males (p< 0.001 according to the log-rank test) (Figure 2). In the whole study cohort, the medication class discontinued the earliest was most often statins (n = 750), followed by antiplatelet agents (n = 677).

Table 2 shows the results of the Cox regression analysis of factors associated with the probability of non-persistence. In the whole study cohort, female sex, atrial fibrillation, and being a new user of at least one of the three medication classes were associated with increased probability of non-persistence, while increasing age, history of MI, Parkinson’s disease, increasing overall number of medications, and administration of beta-blockers and mineralocorticoid receptor antagonists were associated with persistence. Among males, university education, atrial fibrillation, and epilepsy were associated with non-persistence, while history of ischemic stroke or MI, an increasing overall number of medications, and general practitioner as index prescriber of at least one of the analysed medication classes appeared as factors associated with persistence. Among females, being a new user of at least one of the three medication classes was associated with an increased likelihood of non-persistence, while increasing age was associated with persistence. The value of Harrell´s C-index for the Cox model was determined as 0.599 for the whole study cohort; 0.633 and 0.587 for male and female groups, respectively.

**TABLE 2:** Multivariate analysis of the association between patient- and medication-related characteristics and the probability of non-persistence in the whole study cohort and in the groups of males and females.

Factor	The whole study cohort (n = 3,401)	Males (n = 1,548)	Females (n = 1,853)
Socio-demographic characteristics			
Age	**0.99 (0.98–0.99)**	1.01 (0.99–1.02)	**0.98 (0.97–0.99)**
Female sex	**1.34 (1.21–1.48)**		
University education	1.16 (0.98–1.38)	**1.26 (1.03–1.55)**	0.89 (0.63–1.26)
Employed patients	0.97 (0.78–1.20)	1.00 (0.77–1.31)	0.95 (0.66–1.37)
History of CV events^a^			
History of ischemic stroke	0.95 (0.84–1.08)	**0.78 (0.64–0.95)**	1.09 (0.93–1.29)
History of TIA	1.08 (0.90–1.28)	1.10 (0.81–1.50)	1.06 (0.85–1.32)
History of MI	**0.77 (0.64–0.94)**	**0.71 (0.53–0.96)**	0.83 (0.64–1.07)
Comorbid conditions			
Number of comorbid conditions	0.97 (0.88–1.07)	0.99 (0.86–1.15)	0.96 (0.84–1.09)
Chronic heart failure	1.13 (0.91–1.42)	1.16 (0.80–1.68)	1.18 (0.88–1.56)
Atrial fibrillation	**1.24 (1.02–1.51)**	**1.40 (1.04–1.89)**	1.12 (0.86–1.46)
Diabetes mellitus	0.87 (0.76–1.00)	0.93 (0.75–1.14)	0.86 (0.71–1.04)
Hypercholesterolemia	0.98 (0.85–1.13)	0.82 (0.66–1.01)	1.09 (0.91–1.32)
Dementia	0.92 (0.74–1.14)	0.81 (0.55–1.19)	0.96 (0.72–1.26)
Depression	1.03 (0.86–1.24)	0.91 (0.64–1.30)	1.08 (0.87–1.35)
Anxiety disorders	1.12 (0.97–1.30)	1.03 (0.81–1.32)	1.17 (0.96–1.42)
Parkinson’s disease	**0.75 (0.57–0.99)**	0.78 (0.48–1.26)	0.76 (0.54–1.07)
Epilepsy	1.27 (0.95–1.71)	**1.68 (1.11–2.53)**	1.05 (0.68–1.62)
Bronchial asthma/COPD	1.09 (0.93–1.27)	0.94 (0.74–1.21)	1.19 (0.96–1.46)
CV co-medication			
Number of medications	**0.94 (0.92–0.97)**	**0.92 (0.88–0.95)**	0.98 (0.94–1.01)
Number of CV medications	1.03 (0.99–1.08)	1.00 (0.93–1.09)	1.04 (0.98–1.10)
Cardiac glycosides	0.95 (0.75–1.21)	0.98 (0.62–1.56)	0.99 (0.74–1.32)
Antiarrhythmic agents	1.07 (0.86–1.33)	1.14 (0.82–1.61)	1.06 (0.80–1.42)
Beta-blockers	**0.85 (0.75–0.98)**	0.87 (0.69–1.09)	0.88 (0.74–1.04)
Loop diuretics	1.01 (0.87–1.17)	0.95 (0.73–1.23)	1.01 (0.85–1.21)
Thiazide diuretics	0.99 (0.87–1.12)	0.97 (0.79–1.19)	0.98 (0.84–1.15)
Mineralocorticoid receptor antagonists	**0.76 (0.60–0.96)**	0.71 (0.49–1.04)	0.81 (0.60–1.11)
Calcium channel blockers	0.97 (0.86–1.10)	1.07 (0.88–1.31)	0.92 (0.79–1.07)
Anticoagulants	0.92 (0.80–1.05)	1.01 (0.82–1.26)	0.90 (0.76–1.07)
Lipid-lowering agents other than statins^b^	1.00 (0.84–1.17)	0.85 (0.65–1.12)	1.11 (0.90–1.36)
Study medication-related characteristics			
Patient´s co-payment (EUR)^c^	1.00 (0.99–1.02)	1.00 (0.99–1.02)	1.00 (0.99–1.02)
New user^d^	**1.17 (1.03–1.34)**	0.96 (0.78–1.18)	**1.36 (1.14–1.61)**
General practitioner as index prescriber^e^	0.96 (0.81–1.14)	**0.74 (0.58–0.96)**	1.21 (0.95–1.54)

Values represent hazard ratios (95% confidence intervals). In the case of statistical significance (p< 0.05), the values are expressed in bold. CV, cardiovascular; TIA, transient ischemic attack; MI, myocardial infarction; COPD, chronic obstructive pulmonary disease.

^a^ The time period covered by “history” – 5 years before the index date of this study.

^b^ Lipid lowering agents other than statins–ezetimibe and fibrates.

^c^ Co-payment–calculated as a sum of co-payments of particular analysed medications (statins, antiplatelet agents, ACEIs/ARBs) paid by the patient per month.

^d^ New user–patient in whom at least one of the analysed medications (statins, antiplatelet agents, ACEIs/ARBs) was initiated in association with the diagnosis of peripheral arterial disease.

^e^ General practitioner as index prescriber–whether an initial prescriber of any of the analysed medication classes (statins, antiplatelet agents, ACEIs/ARBs) was a general practitioner.

## 4 Discussion

At the end of the five-year follow-up period, non-persistence with at least one of the three analysed medication classes (statins, antiplatelet agents and ACEIs/ARBs) was found in more than half of older patients with PAD in the study cohort. In our previous studies focused on evaluation of non-persistence with individual medication classes, we found lower proportions of non-persistent patients at the end of the 5-year follow-up period: 35.7% of 8,330 statin users (Wawruch et al., 2019), 33.0% of 9,178 users of antiplatelet agents (Wawruch et al., 2021), and 23.2% of 7,080 users of ACEIs/ARBs (Wawruch et al., 2022). This highlights the importance of looking also at the patients’ overall medication used for the treatment of a certain disease. The analysis of persistence/adherence to single medication classes can give inappropriately high estimates of persistence/adherence regarding the treatment of diseases requiring treatment with multiple medications.

More females than males were identified as non-persistent (58.8% vs. 50.3%). There were no factors consistently associated with an increased or decreased probability of non-persistence among females, males and the whole cohort. There were no factors associated with the likelihood of non-persistence consistently among both sexes, either.

Female sex was associated with non-persistence in the whole study cohort. Female sex appeared as a factor associated with non-persistence also in the cohort study by Alfian et al. (2019a) which analysed predictors of non-persistence with antihypertensive drugs among patients taking oral antidiabetic medications. Persistence was lower in women than men in the retrospective cohort study by Koenig et al. (2024) which analysed adherence to and persistence with lipid-lowering therapy in patients with dyslipidaemia. Female sex was also associated with non-persistence in the study by Chen et al. (2019) which analysed long-term adherence to and persistence with statin treatment initiated after discharge from hospital for new onset of atherosclerotic CV disease. According to Venditti et al. (2023), the reasons for sex-related differences in medication adherence are largely unknown. Several factors, e.g., biological, treatment-related, psychosocial, socioeconomic, and mood-related aspects, and their complex interplay, may contribute to these sex-related differences. In comparison with males, females experience adverse drug reactions more frequently, they have lower awareness of their CV risk, harbour more negative perceptions about diseases and treatment, and experience depressive disorders more frequently. That review identified female sex as a risk factor for poorer adherence in patients with type 2 diabetes mellitus or hypercholesterolemia. On the other hand, in the study by Hero et al. (2021), women were less prone to discontinue lipid-lowering therapy at 18 months but not at 36 months. Female sex was associated with persistence also in the study by Tajchman et al. (2021) which evaluated persistence with P2Y_12_ inhibitors in patients with MI. According to the study by Choi et al. (2017), female sex was a positive predictor of persistence with antihypertensive treatment of naive older patients.

In this study, increasing age was associated with persistence in the whole study cohort and among females. This result indicates a careful medication-taking behaviour in older hypertensive PAD patients. Similarly to our study, Tajchman et al. (2021) reported advanced age to be associated with persistence with P2Y_12_ inhibitors. On the other hand, in the study by Alfian et al. (2019a), older age was associated with non-persistence. Older age was an independent predictor of non-persistence also in the cohort study by Kim et al. (2021) which analysed non-persistence with antiplatelet medications in ischemic stroke patients aged ≥75 years. Choi et al. (2017) reported a significantly higher risk for non-persistence with antihypertensive medications in very old patients aged ≥80 years in comparison with older patients aged 65–79 years. Age >75 years was associated with statin non-persistence in the study by Chen et al. (2019). In our study, university education was associated with an increased probability of non-persistence among males. Contrary to this, level of education did not influence the discontinuation of lipid-lowering therapy in the study by Hero et al. (2021).

History of ischemic stroke among males and history of MI in the whole study cohort and among males were associated with persistence. This result may indicate better awareness of the importance of secondary preventive medications in patients after MACE. Diagnosis of acute MI receiving revascularisation as well as coronary heart disease receiving revascularisation were associated with persistence with statins also in the study by Chen et al. (2019).

Among comorbid conditions, atrial fibrillation increased the probability of non-persistence in the whole study cohort and among males. In treatment of atrial fibrillation, anticoagulants are indicated to prevent cardioembolic stroke (Hindricks et al., 2021). The combination of anticoagulants with antiplatelet agents is associated with an increased risk of bleeding. The fear of bleeding among both physicians and patients may be a reason for discontinuation of antiplatelet agents in patients with PAD and atrial fibrillation. Epilepsy appeared as a factor associated with an increased probability of non-persistence among males. In contrast to this finding, epilepsy was associated with persistence in our previous cohort study on non-persistence with antiplatelet agents in older patients after ischaemic stroke (Wawruch et al., 2016).

Increasing overall number of medications was associated with persistence in the whole study cohort and among males. This result may be associated with good persistence in patients with polypharmacy who are used to taking several medications simultaneously. In line with our finding, taking less than four prescribed drugs was associated with non-persistence in the study by Kim et al. (2021) on persistence with antiplatelet therapy among older patients after ischemic stroke.

Similarly to our previous studies on persistence with the individual medication classes (Wawruch et al., 2019; Wawruch et al., 2021; Wawruch et al., 2022), being a new user of at least one of the three medication classes was associated with an increased probability of non-persistence in the whole study cohort and among females. This result may be explained by possible adverse effects or intolerance which may occur at the beginning of treatment. Analogously, prior use of antiplatelet agents and statins was associated with lower probability of discontinuation of antiplatelet agents in the study by Liu et al. (2019) which analysed persistence among patients with acute coronary syndromes.

General practitioner as index prescriber of at least one of the analysed medication classes was associated with persistence among males. This result shows the important role of general practitioners in the management of patients’ adherence to pharmacotherapy. Similarly, family medicine specialty of the prescribing physician was associated with persistence with statins initiated after hospital discharge for atherosclerotic CV disease in a study by Chen et al. (2019).

Administration of beta-blockers and mineralocorticoid receptor antagonists was associated with persistence in the whole study cohort. Similarly, in the study by Tajchman et al. (2021) concomitant pharmacotherapy with ACEIs, statins, beta-blockers, oral anticoagulants, and aspirin was associated with persistence with P2Y_12_ inhibitors. However, that study analysed patients with MI in whom beta-blockers are indicated as part of secondary preventive therapy (Ibanez et al., 2018). On the other hand, beta-blocking agents as initial drug class were associated with non-persistence with antihypertensive drugs in the cohort study by Alfian et al. (2019a). Also, initially chosen antihypertensive medication class other than beta-blockers was a predictor for persistence in the study by Choi et al. (2017).

We found some differences regarding factors associated with non-persistence between the studies in which the medication classes (statins, antiplatelet agents, ACEIs/ARBs) were evaluated separately (Wawruch et al., 2019; Wawruch et al., 2021; Wawruch et al., 2022) and the study described in this manuscript. For example, atrial fibrillation represented a factor associated with persistence in the study focused on users of ACEIs/ARBs, while it was associated with non-persistence in the study focused on users of antiplatelet agents and in this study focused on patients taking a combination of all three medication classes. Parkinson’s disease was associated with persistence only in the present study on combination treatment while in the studies on individual medication classes it was not associated with the likelihood of persistence. On the other hand, increasing number of medications was associated with persistence while being a new user was associated with non-persistence in all three studies on separate medication classes as well as in this study.

Our study has several limitations which should be considered when the study findings are interpreted. Our study is based on data from the database of the General Health Insurance Company which was created for insurance and reimbursement purposes and not for research analyses. It is impossible to identify the reasons for discontinuation and who was responsible for the discontinuation of the medication, whether it was a physician or a patient. These data do not make it possible to determine whether patients really took their medications. The database of the General Health Insurance Company does not include data on the grade and severity of PAD. These characteristics may also influence adherence to treatment. We did not have access to patients’ data beyond the end of the study period. It was therefore impossible to identify any 6-month treatment gaps during less than 6 months before the end of the study follow up. On the other hand, the large sample size as well as the data covering all administrative regions of the Slovak Republic and detailed and precise data on comorbid conditions and medications represent the strengths of our study. Analysis of the three different medication classes taken together represents another strength of this study. These medication classes are all relevant for the treatment of hypertensive PAD patients and it is therefore important to look at the whole combination, in addition to investigating the medication classes separately.

Despite the limitations mentioned above, our study revealed several findings which have important implications for clinical practice. In younger patients, females, those with lower overall number of medications, patients with atrial fibrillation, those without MI or Parkinson’s disease, those without beta-blockers or mineralocorticoid receptor antagonists, and in new users of at least one of the three analysed medication classes, increased attention should be paid to improving their persistence with the combination of medications in order to ensure successful secondary prevention of PAD.

## 5 Conclusion

Our study revealed that over half of older hypertensive PAD patients became non-persistent with at least one of the analysed medication classes (statins, antiplatelet agents, ACEIs/ARBs) during the 5-year follow-up. Significantly higher proportion of females than males was identified as non-persistent. There were differences in factors associated with increased or decreased likelihood of non-persistence between both sex groups and the whole study cohort. More factors were associated with the probability of non-persistence among males in comparison with females. In females, only two factors were determined, namely, increasing age associated with persistence and being a new user associated with non-persistence. Identification of sex differences in factors associated with non-persistence in our study makes it possible to determine the groups of patients who are risky in terms of non-persistence with multiple medications.

## Data Availability

The data analyzed in this study is subject to the following licenses/restrictions: The data that support the findings of this study are available from the General Health Insurance Company but restrictions apply to the availability of these data, which were used under license for the current study, and so are not publicly available. Data are however available from the authors upon reasonable request and with permission of the General Health Insurance Company. Requests to access these datasets should be directed to Martin Wawruch, martin.wawruch@gmail.com.
